# ORC6, a novel prognostic biomarker, correlates with T regulatory cell infiltration in prostate adenocarcinoma: a pan-cancer analysis

**DOI:** 10.1186/s12885-023-10763-z

**Published:** 2023-03-29

**Authors:** Yuan Lin, Ying Zhang, Zhouting Tuo, Liang Gao, Demao Ding, Liangkuan Bi, Dexin Yu, Zhengmei Lv, Jiani Wang, Xin Chen

**Affiliations:** 1 Department of Urology, The Second Affiliated Hospital of Anhui Medical University, Hefei, Anhui China; 2 Center for Clinical Medicine, Huatuo Institute of Medical Innovation (HTIMI), Berlin, Germany; 3 Department of Histology and Embryology, School of Basic Medical Sciences, Anhui Medical University, Anhui, China; 4 School of Health Administration, Anhui Medical University, Hefei, China; 5 Charité-Universitätsmedizin Berlin, Corporate Member of Freie Universität Berlin, Humboldt-Universität Zu Berlin, Berlin, Germany; 6 Berlin Institute of Health, Institute for Social Medicine, Epidemiology and Health Economics, Berlin, Germany

**Keywords:** ORC6, Pan-cancer, Prognosis, Prostate adenocarcinoma, Immunotherapy

## Abstract

**Background:**

The origin recognition complex (ORC), a six-subunit DNA-binding complex, participates in DNA replication in cancer cells. Specifically in prostate cancers, ORC participates the androgen receptor (AR) regulated genomic amplification and tumor proliferation throughout the entire cell cycle. Of note, ORC6, the smallest subunit of ORC, has been reported to be dysregulated in some types of cancers (including prostate cancer), however, its prognostic and immunological significances remain yet to be elucidated.

**Methods:**

In the current study, we comprehensively investigated the potential prognostic and immunological role of ORC6 in 33 human tumors using multiple databases, such as TCGA, Genotype-Tissue Expression, CCLE, UCSC Xena, cBioPortal, Human Protein Atlas, GeneCards, STRING, MSigDB, TISIDB, and TIMER2 databases.

**Results:**

ORC6 expression was significantly upregulated in 29 types of cancers compared to the corresponding normal adjacent tissues. ORC6 overexpression correlated with higher stage and worse prognostic outcomes in most cancer types analyzed. Additionally, ORC6 was involved in the cell cycle pathway, DNA replication, and mismatch repair pathways in most tumor types. A negative correlation was observed between the tumor endothelial cell infiltration and ORC6 expression in almost all tumors, whereas the immune infiltration of T regulatory cell was noted to be statistically positively correlated with the expression of ORC6 in prostate cancer tissues. Furthermore, in most tumor types, immunosuppression-related genes, especially TGFBR1 and PD-L1 (CD274), exhibited a specific correlation with the expression of ORC6.

**Conclusions:**

This comprehensive pan-cancer analysis revealed that *ORC6* expression serves as a prognostic biomarker and that ORC6 is involved in the regulation of various biological pathways, the tumor microenvironment, and the immunosuppression status in several human cancers, suggesting its potential diagnostic, prognostic, and therapeutic value in pan-cancer, especially in prostate adenocarcinoma.

**Supplementary Information:**

The online version contains supplementary material available at 10.1186/s12885-023-10763-z.

## Introduction

The origin recognition complex (ORC) is a six-subunit DNA-binding complex crucial for the initiation of DNA replication in eukaryotes, as its binding to origin sequences triggers the replication process [1]. ORC6 is the smallest subunit of the ORC. Interestingly, ORC6 can bind to DNA independently in human cells, indicating its ORC-independent functions [2, 3]. ORC6 is involved in the tumorigenic process of a limited number of cancer types [4–7]. In colorectal cancer, the ORC6 expression is upregulated, while a lower ORC6 expression correlates with a favorable long-term cancer prognosis, indicating that ORC6 may act as an oncogene in the early stage but exert the suppressor effects in the advanced stage [4]. Furthermore, it has been reported that decreased ORC6 expression may sensitize colon cancer cells to 5-Fluorouracil and cisplatin [6]. In hepatocellular carcinoma, it has been demonstrated that ORC6 may promote the tumor proliferation, migration, and invasion [5].

In prostate cancers, androgen receptor (AR) overexpression allows the cancer cells to advance to androgen castration stages. Prostate cancer cells with AR amplification can endure with androgen deprivation therapies, progressing to castration resistant prostate cancer (CRPC) [8]. Accumulative evidence have showed that, during early G1-phase of the cell cycle, nuclear AR in metastatic CRPC (mCRPC) cells binds to DNA at origins of replication sites (part of the ORC) needed for licensing DNA replication in the S-phase [9, 10]. Also, AR, as a licensing factor, remains to be associated with the ORC during the entire cell cycle progression until the late mitosis phase before its degradation, which allows again relicensing to occur in the next cell cycle [9]. Specifically, ORC6 may also participate in the tumorigenesis, while the detailed function is unclear [11].

Owing to the development of bioinformatic tools, the identification and characterization of novel pan-cancer genes through several public databases, including The Cancer Genome Atlas (TCGA) and Genotype-Tissue Expression (GTEx), become efficient methods to identify new potential drug targets [12–15]. In the current study, we planned to use multiple databases to clarify the landscape of ORC6 status in 33 most common types of cancer and to perform a comprehensive analysis of the influence of ORC6 on prognosis across these cancer types. The relationships between the ORC6 expression and tumor clinical stage, prognostic significance, biological pathways, tumor mutational burden (TMB), microsatellite instability (MSI), expression level of genes related to mismatch repair (MMR), immune subtype, tumor immune cell infiltration, and immune checkpoint genes in diverse cancers (especially prostate cancer) were also investigated.

## Materials and methods

### *ORC6* mRNA expression levels in pan-cancer

We obtained the *ORC6* mRNA expression levels and clinical data of TCGA and GTEx cohorts from the UCSC Xena database (https://xenabrowser.net/datapages/). *p*-values < 0.05 (two-tailed) were regarded as statistically significant (^∗^*p* < 0.05, ^∗∗^*p* < 0.01, ^∗∗∗^*p* < 0.001, and ^∗∗∗∗^*p* < 0.0001). Then, we downloaded the *ORC6* mRNA expression data for diverse cancer cell lines from the Cancer Cell Line Encyclopedia (CCLE) database (https://portals.broadinstitute.org/ccle/data) and the DNA copy number and methylation information from the cBioPortal database (https://www.cbioportal.org/).

### Immunohistochemical (IHC) staining and subcellular localization of ORC6 

We further evaluated the ORC6 protein levels based on the IHC staining data provide by the Human Protein Atlas (HPA) database (https://www.proteinatlas.org/). Subcellular localization of ORC6 was observed in the GeneCards database (https://www.genecards.org/). The data from the STRING database (https://string-db.org/) was built for the protein–protein interaction (PPI) network.

### Prognostic value of *ORC6*

In order to explore the association between *ORC6* expression and prognostic information, Kaplan–Meier analysis of the TCGA datasets was performed. Four survival indicators, including the overall survival (OS), disease-specific survival (DSS), disease-free interval (DFI), and progression-free interval (PFI), were enrolled in the analysis. We set up univariate Cox regression analyses to evaluate the prognostic significance of *ORC6* in predicting these four survival indicators in these 33 types of cancers. The results of the regression analyses are shown using a forest plot.

### Correlation between *ORC6* expression and TMB, MSI, and MMR gene expression

The TMB analysis was conducted with the R package (edgeR) using the human pan-cancer somatic data (MAF data) from TCGA database. The MSI score was used as per a published study [16]. Both TMB and MSI were calculated using the Pearson’s method. The “Gene_Corr” module of TIMER2 was utilized to analyze MMR gene expression levels in the TCGA database. Five important MMR genes, including MutL protein homolog 1 (*MLH1*), MutS protein homolog 2 (*MSH2*), MutS homologue 6 (*MSH6*), epithelial cell adhesion molecular (*EPCAM*), and PMS1 homolog 2 (*PMS2*) were selected for the correlation analysis. The correlation degree was calculated with purity-adjusted Spearman and plotted in a heatmap.

### Functional enrichment analysis of ORC6 across cancers 

First, we used the data from the TCGA database to explore the potential biological and molecular functions of ORC6 via both the Gene Set Enrichment Analysis (GSEA) and Gene Set Variation Analysis (GSVA). The Kyoto Encyclopedia of Genes and Genomes (KEGG) pathway database was selected for GSEA enrichment analyses with the R package “clusterProfiler” [17–20]. Then, we screened and demonstrated the top 20 most significant positive correlated pathways. In addition, we performed the GSVA with the R package “GSVA” using hallmark pathways from the MSigDB database (https://www.gsea-msigdb.org/gsea/msigdb/index.jsp).

### Immune association analysis of *ORC6*

The *ORC6* expression stratified by the immune subtypes across cancers was investigated in the TISIDB database (http://cis.hku.hk/TISIDB/). The influence of *ORC6* expression on immune cell infiltration was analyzed using the datasets from the TIMER2 database (http://timer.cistrome.org/). Cancer-associated fibroblasts, tumor endothelial cells, T regulatory (Treg) cells, and CD8^+^ T cells were selected for detailed analysis. The EPIC, MCP-counter, xCELL, CIBERSORT, CIBERSORT-ABS, quanTIseq, and TIMER algorithms were utilized to evaluate endemic tumor cell types in TCGA. The Spearman correlation analysis between the *ORC6* expression and immune checkpoint-associated genes was conducted using the TCGA pan-cancer data and visualized using a heatmap.

### IHC analysis of ORC6, FOXP3 and CD4 expressions in prostate cancer

A total of 19 formalin-fixed and paraffin-embedded prostate adenocarcinoma tumor tissue samples were rehydrated and incubated with the anti-ORC6 (1:400; Genetex, USA), anti-FOXP3 (1:400; Genetex, USA), and anti-CD4 (1:400; Thermo Fisher Scientific, USA) antibodies in a humid box at 4 °C. Three representative 500 × 430 µm areas with more than 50% tumor cell and more than 30 CD4 + cells were enrolled for staining analysis. The expression level of ORC6 was evaluated based on the tissue immunostaining score (TIS), which was defined as the product of the intensity score (IS) and quantity score (QS) (i.e., TIS = IS × QS). The positive staining of ORC6 in tumor cells and FOXP3 in CD4 + cells was regarded as the staining percentage. The staining percentage scores and the staining intensity scores were calculated as previously published [21]. For the FOXP3 and CD4, positive staining cells were counted for the Treg and CD4 + T cells. Mann–Whitney U test was applied to calculate the relationship between the ORC6 expression and Treg and CD4 + T cell number with the GraphPad Prism 8 (GraphPad Software, USA). All *p*-values < 0.05 (two-tailed) were regarded as statistically significant, and denoted as ∗ *p* < 0.05, ∗  ∗ *p* < 0.01, ∗  ∗  ∗ *p* < 0.001, and ∗  ∗  ∗  ∗ *p* < 0.0001, respectively. We obtained written informed consent from all patients and implemented all procedures under the Declaration of Helsinki.

## Results

### *ORC6* mRNA expression in pan-cancer

**Fig. 1:**
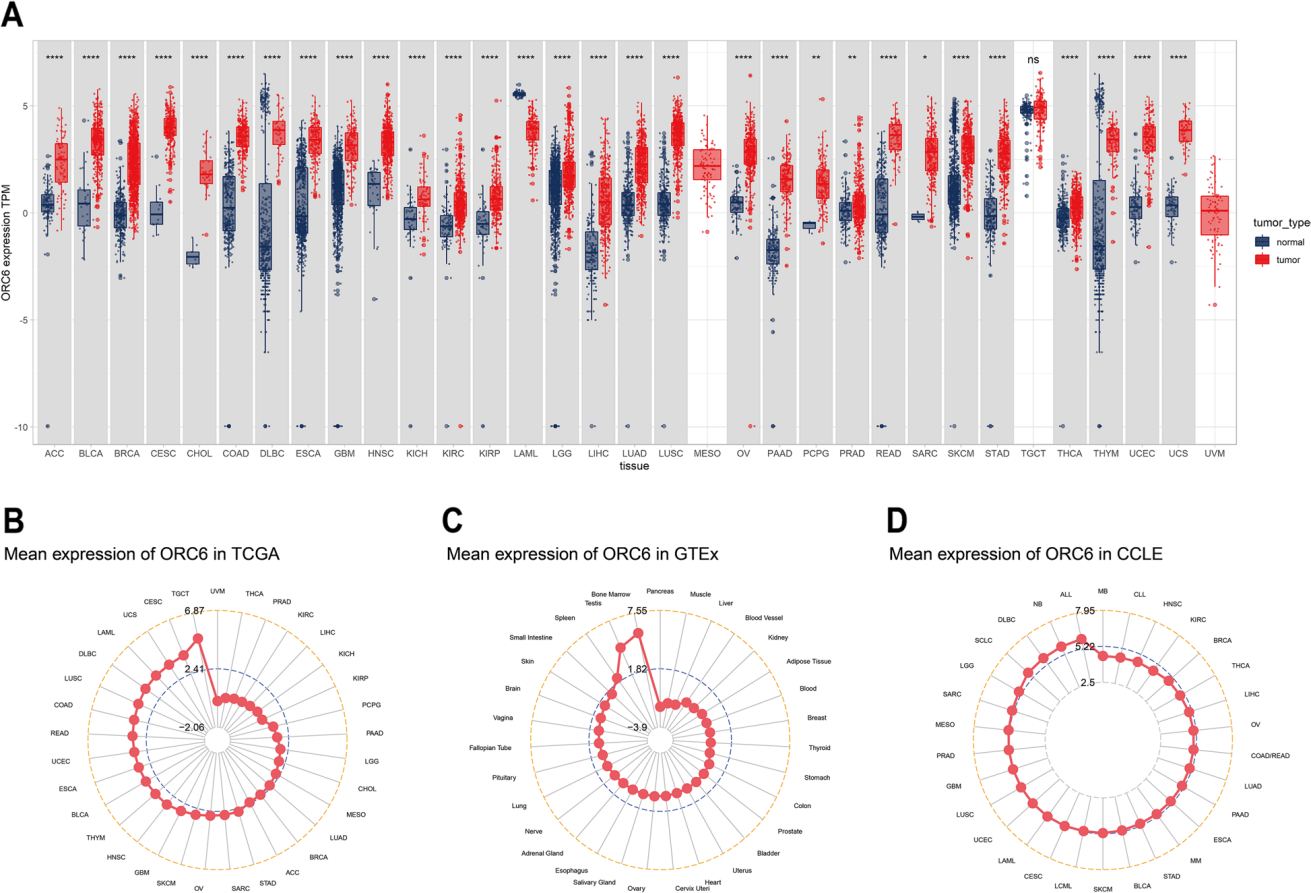
Pan-cancer *ORC6* mRNA expression level. **A** *ORC6* mRNA expression across cancers. **B** Mean *ORC6* mRNA expression level in tumor tissues from TCGA database. **C** Mean *ORC6* expression in normal tissues from GTEx database. **D** Mean *ORC6* mRNA expression in tumor cell lines from the Cancer Cell Line Encyclopedia database (CCLE) database. *p < 0.05, **p < 0.01, ****p < 0.0001, ns: not significant

**Fig. 2:**
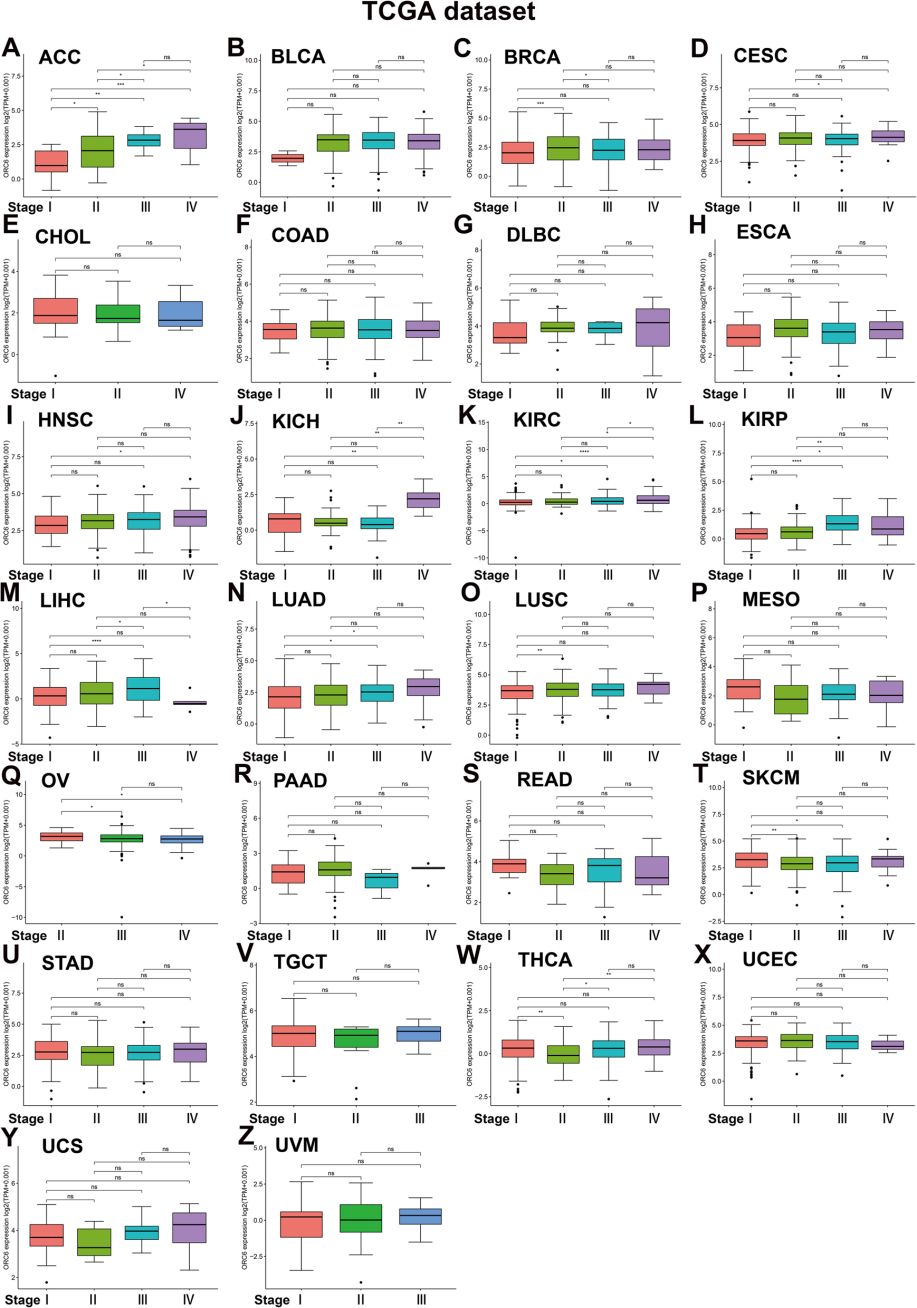
*ORC6* mRNA expression level in diverse tumor stages. **A–Z** *ORC6* mRNA expression level in diverse stages of indicated tumor types from TCGA database. *p < 0.05, **p < 0.01, ***p < 0.001, ****p < 0.0001, ns: not significant

We first investigated the *ORC6* status in pan-cancer by analyzing the available data from TCGA, encompassing 33 most common types of cancers, including BLCA, CESE, and DLBC (Table 1). The increased ORC6 expression was observed in 29 types of cancers, including BLCA, CESE, and PRAD, compared to the corresponding normal adjacent tissues while decreased ORC6 expression was observed only in LAML (Fig. 1A). The highest *ORC6* expression levels were found in TGCT, CESC, and UCS (Fig. 1B). According to the *ORC6* expression levels in normal human tissues based on the GTEx database, *ORC6* was mainly expressed in the bone marrow, testis, and spleen (Fig. 1C). According to the information about distinct cell lines extracted from the CCLE database, the highest *ORC6* expression levels were found in ALL (acute lymphoblastic leukemia), normal breast (NB), and DLBC cells (Fig. 1D). Further analysis of TCGA data revealed that *ORC6* expression was significantly increased in tumor tissues *versus* adjacent normal tissues in 18 types of cancers, including BLCA, BRCA, and PRAD (Supplementary Fig. 1). Further comparison of the *ORC6 *expression according to the TCGA database revealed that *ORC6* expression was significantly increased in higher-stage tumor tissues than in lower-stage tumor tissues in 11 types of cancers, including ACC, KICH, and LUAD; however, it was decreased in OV and SKCM tissues (Fig. 2).

**Table 1:** Abbreviations of 33 cancer types

Cancer name	Abbreviations
Adrenocortical carcinoma	ACC
Bladder urothelial carcinoma	BLCA
Breast invasive carcinoma	BRCA
Cervical squamous cell carcinoma	CESC
Cholangiocarcinoma	CHOL
Colon adenocarcinoma	COAD
Lymphoid neoplasm diffuse large B-cell lymphoma	DLBC
Esophageal carcinoma	ESCA
Glioblastoma	GBM
Head and neck squamous cell carcinoma	HNSC
Kidney chromophobe	KICH
Kidney renal clear cell carcinoma	KIRC
Kidney renal papillary cell carcinoma	KIRP
Acute myeloid leukemia	LAML
Brain lower grade glioma	LGG
Liver hepatocellular carcinoma	LIHC
Lung adenocarcinoma	LUAD
Lung squamous cell carcinoma	LUSC
Mesothelioma	MESO
Ovarian serous cystadenocarcinoma	OV
Pancreatic adenocarcinoma	PAAD
Pheochromocytoma and paraganglioma	PCPG
Prostate adenocarcinoma	PRAD
Rectum adenocarcinoma	READ
Sarcoma	SARC
Skin cutaneous melanoma	SKCM
Stomach adenocarcinoma	STAD
Testicular germ cell tumors	TGCT
Thyroid carcinoma	THCA
Thymoma	THYM
Uterine corpus endometrial carcinoma	UCEC
Uterine carcinosarcoma	UCS
Uveal melanoma	UVM

### Genetic alterations of *ORC6* in pan-cancer

Genetic alterations in *ORC6* were investigated in the cBioPortal database. Patients with PRAD and SARC harboured a high frequency of gene alterations, among which gene amplification was most commonly observed (Supplementary Fig. 2A). Additionally, the *ORC6* mRNA expression level was positively correlated with copy number alteration (CNA) in 21 types of cancers, including BRCA, PRAD, and UCS (Supplementary Fig. 2B). Moreover, the DNA methylation level of the *ORC6* promoter was negatively correlated with *ORC6* mRNA expression level in DLBC, ESCA, PCPG, PRAD, TGCT, THCA, and UCS (Supplementary Fig. 2C).

### Protein expression level and subcellular localization of *ORC6*

Data regarding ORC6 protein levels in various tumors and normal tissues were obtained from the HPA database (Supplementary Fig. 3). The ORC6 protein level was highest in head and neck cancer and testis cancer but lowest in renal cancer (Supplementary Fig. 3A). In normal tissues, the ORC6 was overexpressed in the bone marrow, lymph node, stomach, duodenum, tonsil, colon, pancreas, and testis (Supplementary Fig. 3B). Typical IHC staining figures of ORC6 in 17 pairs of tumors (including BLCA, BRCA, and PRAD) and corresponding normal tissues were shown in Supplementary Fig. 4. Tissues of normal bladder, breast, cervix, colon, oral tissue, kidney, cerebral cortex, liver, lung, ovary, pancreas, prostate, rectum, stomach, testis, thyroid, and endometrium had negative or moderate ORC6 IHC staining, while the corresponding tumor tissues had moderate or strong staining. These results were consistent with the results of ORC6 mRNA expression data from the TCGA database. The ORC6 was mainly located in the nucleus (Supplementary Fig. 3C). Additionally, the PPI network analysis using the tool STRING identified that ORC6 closely interacted with ORC1-5, CDT1, CDC6, MCM4, MCM5, and MCM7 (Supplementary Fig. 3D).

### Prognostic significance of *ORC6* in pan-cancer 

**Fig. 3:**
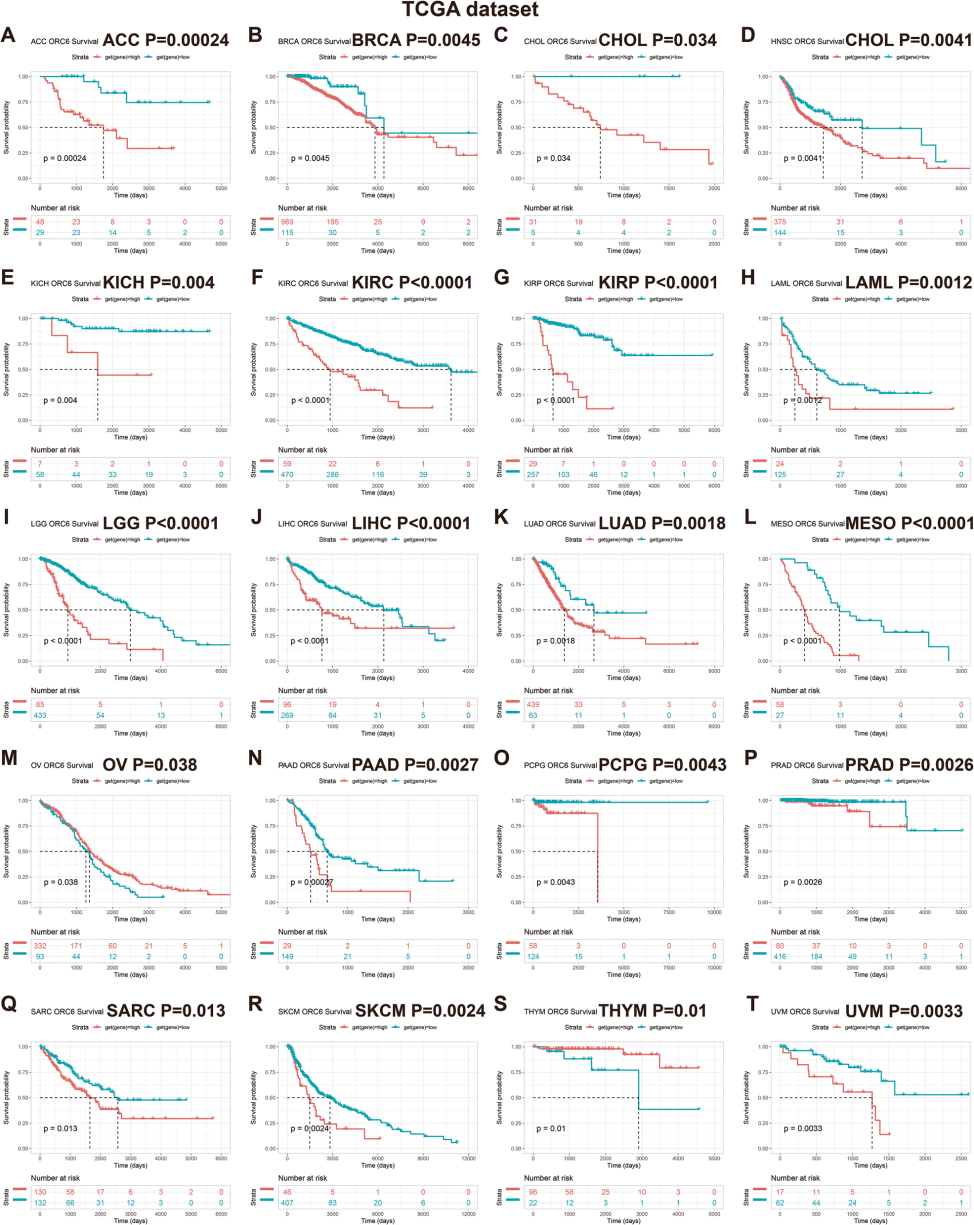
Overall survival (OS) Analysis dependent on *ORC6* expression. **A–T** Kaplan–Meier curves of OS in diverse types of cancers from TCGA database. Statistically non-significant results are not shown

Subsequently, we estimated the survival indicators, including OS, DSS, DFI, and PFI. The OS analysis demonstrated that *ORC6* expression level was as an unfavorable indicator for patients with 18 types of cancers (e.g., KIRC, KIRP and PRAD), and a protective marker only for patients with OV and THYM (Fig. 3). Higher expression of *ORC6* was significantly associated with worse prognosis in DSS for patients with 17 types of cancers (e.g., ACC, LGG and PRAD), while lower expression of *ORC6* was only negatively correlated with the prognosis of COAD, OV, and THYM (Supplementary Fig. 5). According to DFI analysis, *ORC6* high expression level was as an unfavorable indicator for patients with BRCA, COAD, KIRP, LIHC, LUAD, PAAD, PRAD, SARC, and THCA, and a protective marker for patients with OV (Supplementary Fig. 6). Finally, according to PFI analysis, *ORC6* high expression level acted as an unfavorable indicator for patients with 23 types of cancers (e.g., KIRP, LIHC and PRAD), and as a protective marker only in patients with GBM, OV, STAD, and THYM (Supplementary Fig. 7).

**Fig. 4:**
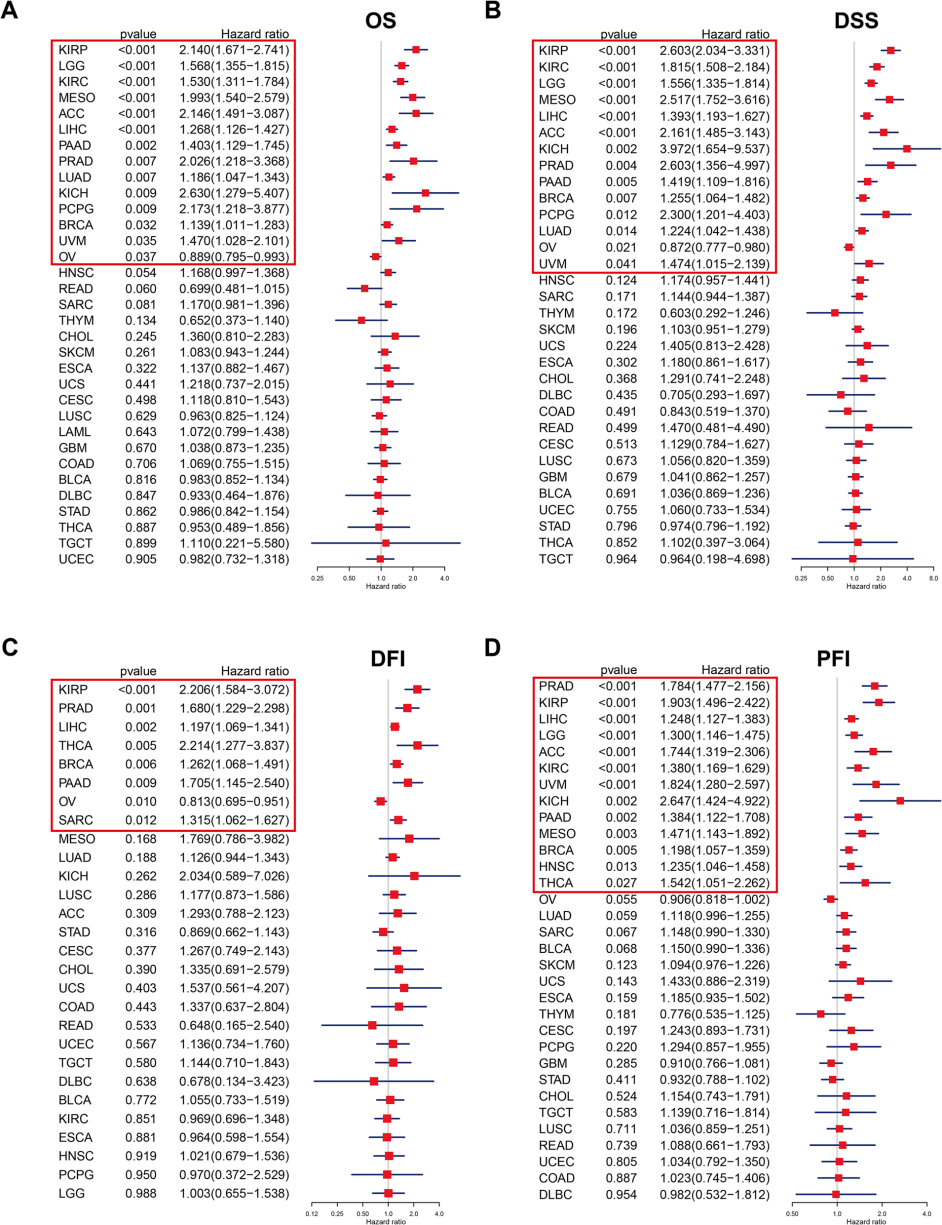
Univariate Cox regression analysis of *ORC6*.** A** Forest plot demonstrated the hazard ratios of OS correlated with *ORC6* expression in diverse types of cancers from TCGA database. **B** Forest plot demonstrated the hazard ratios of DSS correlated with *ORC6* expression in diverse types of cancers from TCGA database. **C** Forest plot demonstrated the hazard ratios of DFI correlated with *ORC6* expression in diverse types of cancers from TCGA database.** D** Forest plot demonstrated the hazard ratios of PFI correlated with *ORC6* expression in diverse types of cancers from TCGA database. The red frame highlights the significant results

To further explore the influence of ORC6 on OS, the univariate Cox regression analysis was performed. The results indicated that high *ORC6* expression level was simultaneously associated with low OS and DSS in 13 types of cancers (e.g., KIRC, KIRP and PRAD), while low *ORC6* expression was associated with low OS and DSS in patients with OV (Fig. 4A-B). Based on DFI analysis, high *ORC6* expression was as an unfavorable indicator for patients with BRCA, KIRP, LIHC, PAAD, PRAD, SARC, and THCA, but a protective marker in OV (Fig. 4C). Finally, the high expression level of *ORC6* was associated with a decreased PFI in 13 types of cancers, including KIRP, LIHC, and PRAD (Fig. 4D). In summary, *ORC6* significantly influenced all survival metrics of only five types of cancers (i.e., BRCA, KIRP, LIHC, PAAD, and PRAD).

### Evaluation of *ORC6* expression and TMB, MSI, and MMR genes expression 

**Fig. 5:**
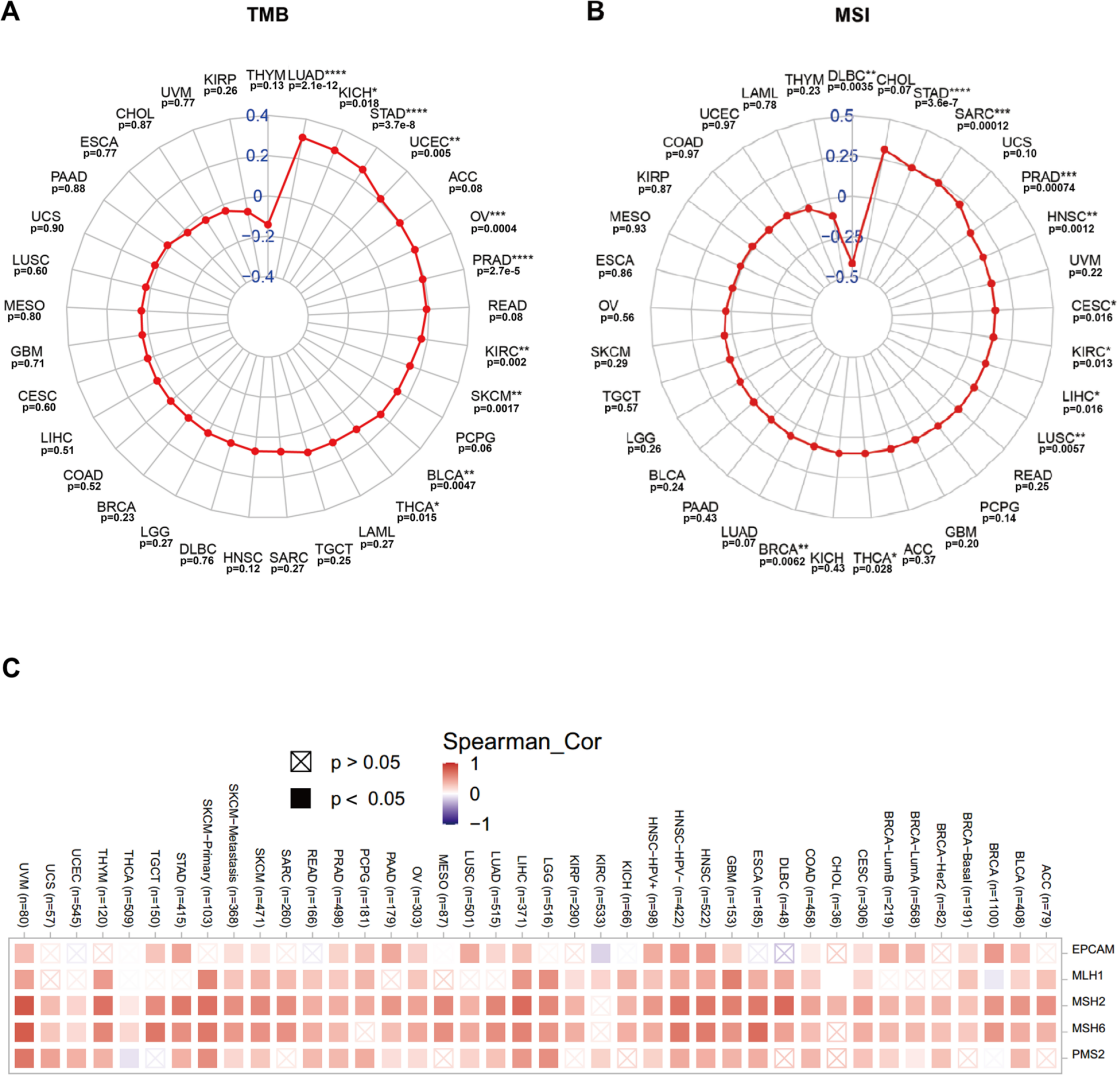
Tumor mutation burden (TMB) and microsatellite instability (MSI) analysis of *ORC6* expression level across cancers. **A** TMB analysis of *ORC6* expression level in 33 TCGA tumor types. **B** MSI analysis of *ORC6* expression in 33 TCGA tumor types. *p < 0.05, **p < 0.01, ***p < 0.001, ****p < 0.0001. **C** Correlation between five MMR gene expression with ORC6 expression level in various tumor types

*ORC6* expression level was positively correlated with TMB in 10 types of cancers, including LUAD, PRAD and STAD (Fig. 5A). Additionally, it was significantly positively correlated with MSI in 10 types of cancers, including PRAD, SARC and STAD, but negatively correlated with MSI in DLBC (Fig. 5B). Five MMR gene expression levels were significantly positively correlated with *ORC6* expression level in most cancers analyzed (e.g., *MLH1*: 65.6%; *MSH2*: 96.9%; *MSH6*: 90.6%; *PMS2*: 71.9%; *EPCAM:* 62.5%) (Fig. 5C).

### GSEA and GSVA of ORC6 

**Fig. 6:**
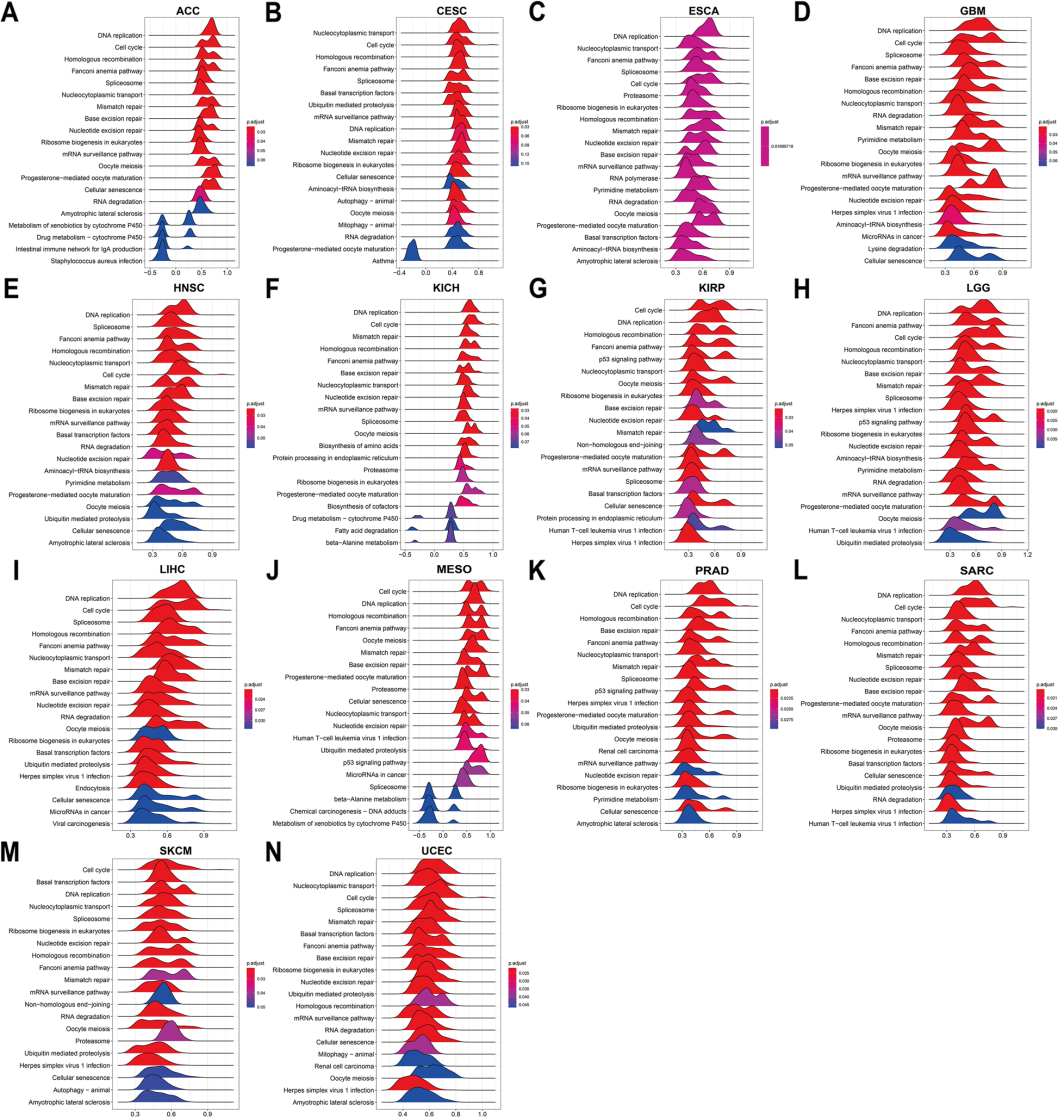
Gene Set Enrichment Analysis (GSEA) of ORC6 across cancers. **A-N** KEGG results of ORC6 GSEA in indicated tumor types using pan-cancer data from TCGA

**Fig. 7:**
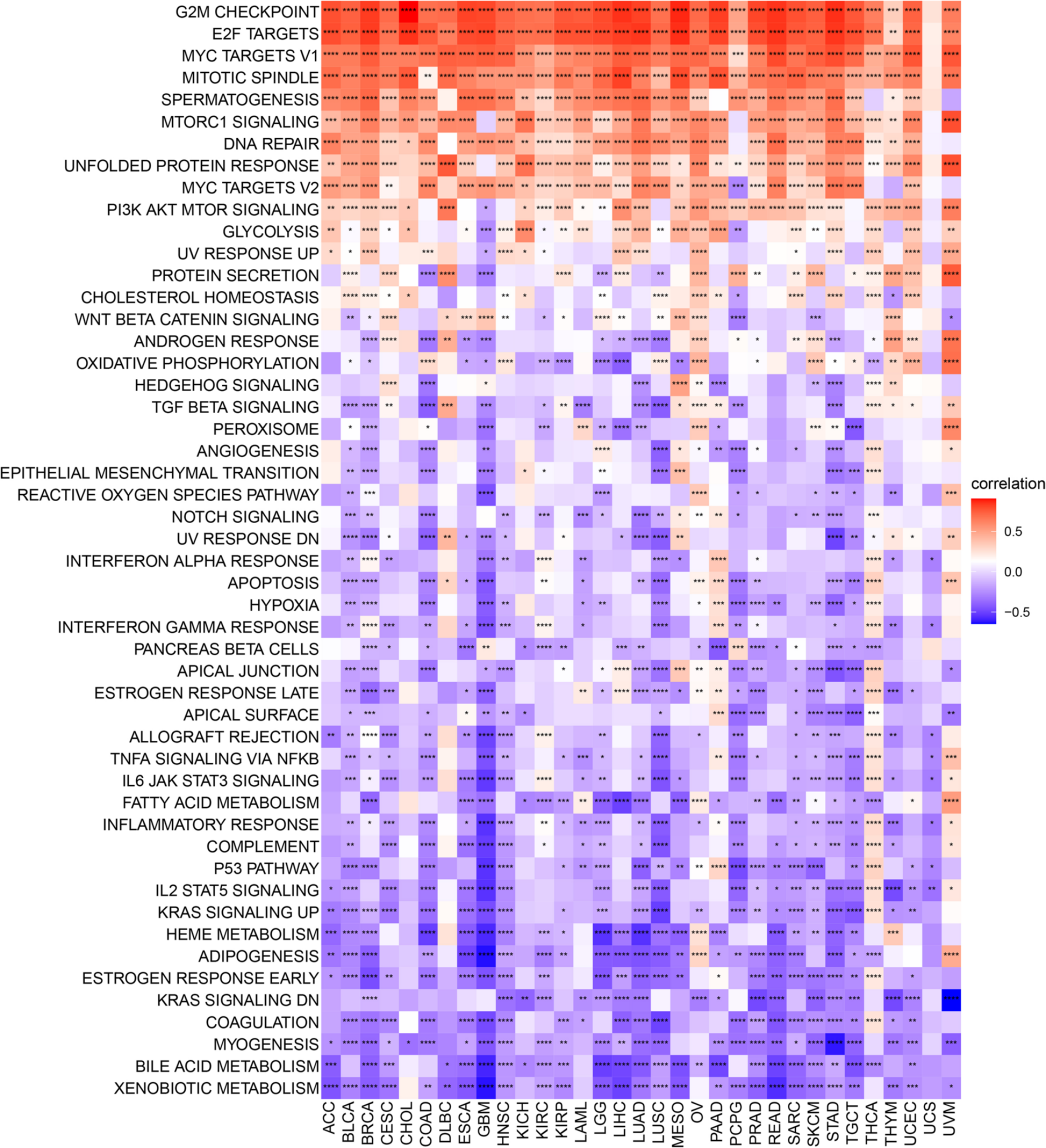
Gene Set Variation Analysis (GSVA) of ORC6 across cancers in MSigDB database

The potential biological pathways associated with ORC6 was predicted through the KEGG pathway analysis, and the top 20 pathways are shown in Fig. 6 and Supplementary Fig. 8. The high *ORC6* expression was significantly associated with the cell cycle and DNA replication related pathways (Fig. 6 and Supplementary Fig. 8). It is noteworthy that ORC6 was also involved in the MMR pathway in 14 types of cancers, including ESCA, GBM and PRAD (Fig. 6). These results indicate a potential role of ORC6 in adjusting the tumor microenvironment. The GSVA score revealed that ORC6 was positively correlated with some cell proliferation pathways (e.g., G2M checkpoint, E2F targets and MYC targets v1–2), “DNA Repair” pathway and “unfolded protein response” pathway in almost all cancers (Fig. 7). These pathways have been identified to be correlated with the advanced stage of cancers and may benefit from immunotherapy [22, 23]. In addition, ORC6 was negatively correlated with several immune pathways (e.g., IL2-STAT5 signaling, inflammatory response and IL6-JAK-STAT3 signaling) in the majority of cancers (Fig. 7).

### Correlations of *ORC6* expression with immune characteristics

Six immune subtypes (e.g., C1: wound healing; C2: IFN-γ dominant; C3: inflammatory; C4: lymphocyte depleted; C5: immunologically quiet; C6: TGF-β-dominant) presented significantly different *ORC6* expression levels in 15 types of cancers, including BRCA, LIHC, LUAD and PRAD (Supplementary Fig. 9). No differences in *ORC6* expression levels in immune cells were observed in the other types of cancers.

**Fig. 8:**
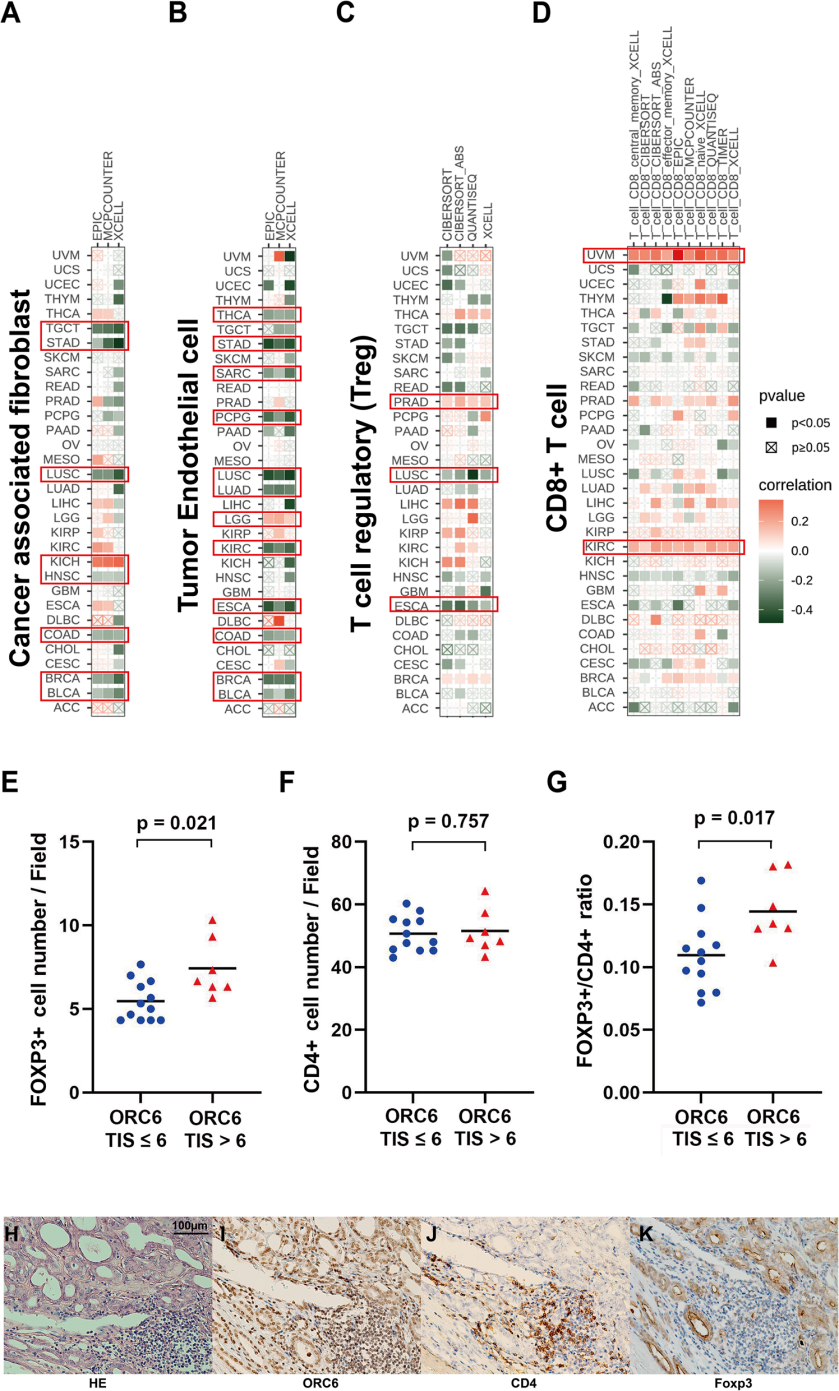
Association between *ORC6* expression and cancer-associated fibroblast, tumor endothelial cell, T regulatory (Treg) cell, and CD8 + T cell infiltration. **A** Association between *ORC6* expression and cancer-associated fibroblast infiltration using TIMER2 database.** B** Association between *ORC6* expression and tumor endothelial cell infiltration using TIMER2 database.** C** Association between *ORC6* expression level and Treg cell infiltration using TIMER2 database.** D** Association between *ORC6* expression level and CD8 + T cell infiltration using TIMER2 database. Red frame highlights the significant results. **E** The association between the *ORC6* expression and FOXP3 + cell number based on immunohistochemical (IHC) results. **F** The association between the expression of *ORC6* and CD4 + T cell number based on immunohistochemical (IHC) results.** G** The association between the ORC6 expression and FOXP3 + /CD4 + ratio based on immunohistochemical (IHC) results. **H** Hematoxylin and eosin staining of prostate adenocarcinoma tissue. **I** IHC staining of ORC6 in prostate adenocarcinoma tumor tissue. **J** IHC staining of CD4 in prostate adenocarcinoma cancer tissue.** K** IHC staining of FOXP3 in CD4 + T cells which were infiltrated in prostate adenocarcinoma cancer tissue

According to the TCGA database, the *ORC6* expression level was inversely related to the infiltration level of cancer-related fibroblasts in seven types of cancers, including BRCA, LUSC, STAD, and TGCT (Fig. 8A). In contrast, a negative correlation was found in KICH (Fig. 8A). Additionally, *ORC6* expression level was negatively correlated with tumor endothelial cell infiltration in 11 types of cancers, including ESCA, KIRC, LUAD, LUSC and STAD, while a positive correlation was observed in LGG (Fig. 8B). Furthermore, a negative correlation was detected between *ORC6* expression level and Treg cell infiltration in ESCA and LUSC, whereas a positive correlation was observed in PRAD (Fig. 8C). Finally, a positive correlation was found between *ORC6* expression level and CD8 + T cell infiltration in KIRC and UVM (Fig. 8D). Furthermore, to validate the correlation between ORC6 expression and Treg cell infiltration, IHC staining of FOXP3, which serves as a lineage specification factor of Treg cells, was performed in tumor samples from 19 patients with prostate adenocarcinoma. Since the median TIS of ORC6 was 6, we stratified the patients into ORC6 TIS ≤ 6 group and TIS > 6 group. The number of Treg in ORC6 TIS > 6 group is more than that of ORC6 TIS ≤ 6 group (mean 7.43 vs 5.47, *p* = 0.021, Fig. 8E), while there is no difference of CD4 + T cell numbers between the two groups (mean 51.57 vs 50.69, *p* = 0.757, Fig. 8F). Of note, the FOXP3 + /CD4 + ratio was also higher in ORC6 TIS > 6 group than that of ORC6 TIS ≤ 6 group (mean 0.14 vs 0.11, *p* = 0.757, Fig. 8G). Representative staining images are shown in Fig. 8H–K.

**Fig. 9:**
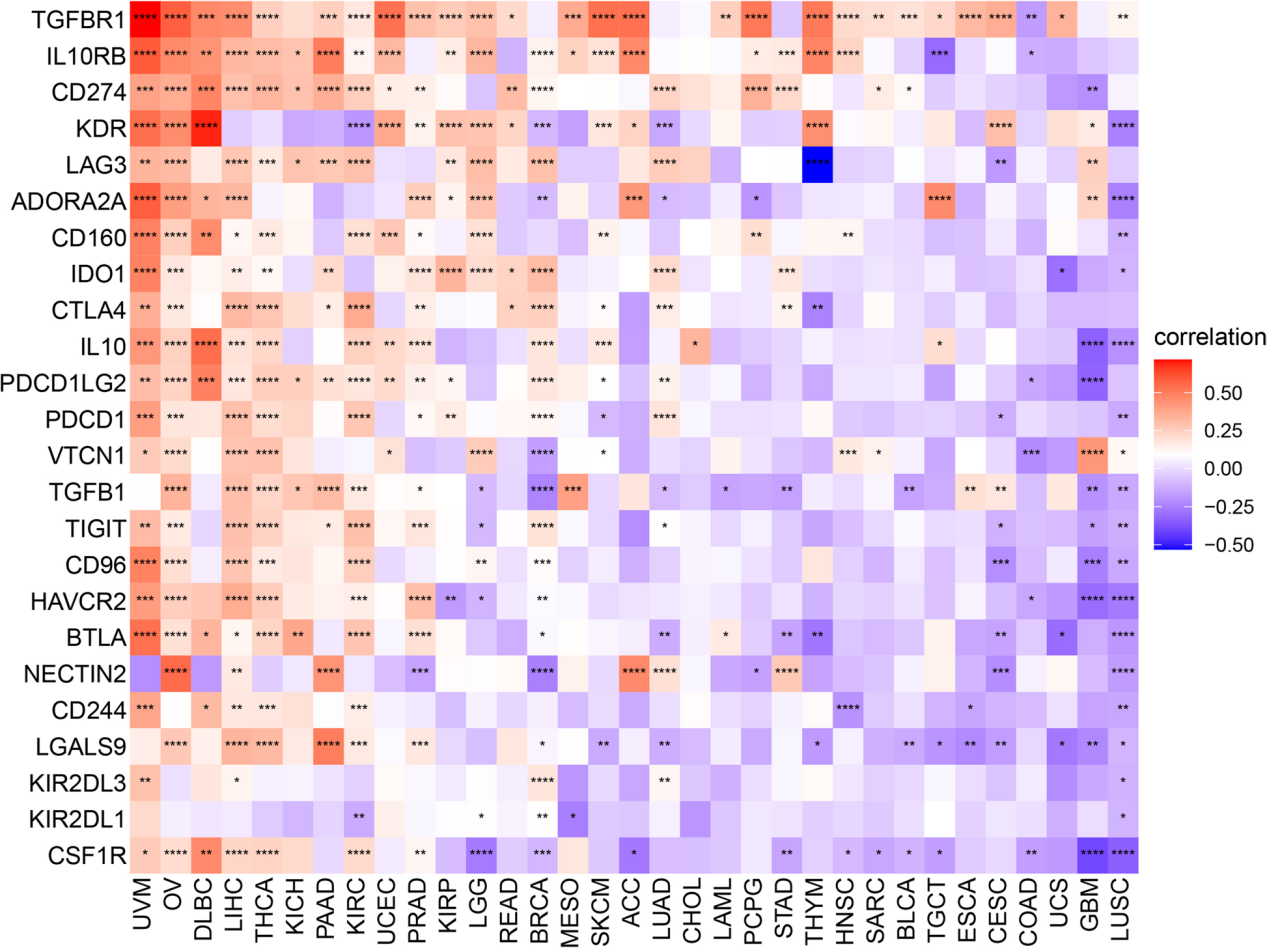
Association between *ORC6* expression and immunosuppressive genes across cancers in The Cancer Genome Atlas

The association between *ORC6* expression and immune related genes expression levels was then evaluated. *ORC6* expression level was significantly correlated with the expression level of a majority of immunosuppressive and immunostimulatory markers in BRCA, DLBC, KIRC, LIHC, LUSC, OV, PAAD, PRAD, THCA, and UVM (Fig. 9 and Supplementary Fig. 10). Interestingly, in most tumor types, immunosuppression-related genes, especially TGFBR1 and PD-L1 (CD274), exhibited a specific correlation with the ORC6 expression (Fig. 9). Altogether, these results indicate that ORC6 may promote immunosuppression in a wide array of cancer types.

## Discussion

Previous studies have demonstrated that ORC6 is involved in a range of biological events during tumor progression [24, 25]. However, the prognostic value of ORC6 expression levels and its potential effect on processes related to tumor development, such as the regulation of the tumor microenvironment and immunosuppression, in a number of human cancers remain unknown and require further study. As far as we know, this is the first comprehensive analysis of the expression and biological function of ORC6 from a pan-cancer perspective.

The pan-cancer analysis demonstrated that *ORC6* expression was significantly upregulated in 29 types of cancers, including BLCA, CESE and PRAD. Analysis of ORC6 protein levels using IHC staining results revealed similar results, confirming that ORC6 broadly participates in the tumorigenesis of different types of cancers. Previous studies demonstrated that ORC6 may irreplaceably promote the cell proliferation through coordinating chromosome replication and segregation with cytokinesis [26, 27]. Interestingly, we found that ORC6 plays multifaceted roles during tumorigenesis, inhibiting or promoting tumor progression depending on the specific types of cancers. The *ORC6* overexpression is correlated with worse prognostic outcomes in the majority of cancers (e.g., KIRC, LIHC, and PRAD) whereas it correlated with a better prognosis in COAD, OV and THYM. Several previous studies also verified our results [4, 7, 27]. The atypical correlation between *ORC6* overexpression and prognosis in OV may be attributed to the fact that *ORC6* is under-expressed exists in the higher stage of OV tumor tissues compared to the lower stage. Altogether, these results imply that *ORC6* expression level may predict the prognosis of cancer patients. Nonetheless, the precise molecular mechanism of action of ORC6 in these cancers remains to be elucidated.

The TMB represents the number of somatic gene mutations existed in the cancer cells [28]. MSI refers to genetic instability caused by impaired DNA MMR [29]. MMR maintains the integrity and stability of the whole genome by correcting DNA replication or recombination errors [30]. Several studies have identified that both TMB and MSI can be useful predictive biomarkers for response to immunotherapy [31–35]. Additionally, MMR deficiency is a sensitive predictor of anti-PD-1/PD-L1 immunotherapy efficacy in multiple cancers [36]. Our study revealed that *ORC6* expression level was closely related with TMB in 10 types of cancers (e.g., LUAD, PRAD and STAD), with MSI in 11 types of cancers (e.g., PRAD, SARC and STAD), and with the expression of 5 MMR genes in a majority of cancers (e.g., HNSC, LIHC and PRAD). Our data showed that GSEA demonstrated a strong correlation between ORC6 and MMR pathways in 14 types of cancers (e.g., PRAD, STAD, and KIRC). Therefore, ORC6 might be a potential therapeutic marker for immunotherapy response. The development of immunotherapy has permitted to greatly improve the perspective of cancer patients at an advanced stage of cancer in recent years [37–40]. Nonetheless, the success of immunotherapy is influenced and sometimes compromised due to tumor-immune system interaction [41]. Our data showed that *ORC6* expression level was significantly related to different immune subtypes in 15 types of cancers (e.g., BRCA, LIHC, and PRAD); these data may partially explain why ORC6 plays different roles in the prognosis and immunotherapy response of diverse cancers.

Accumulative evidence have showed that, immune microenvironment is significantly associated with tumor prognosis[42, 43]. Immune cell infiltration is considered to be an indicator of the immune microenvironment within tumors [44–46]. We report herein for the first time a statistical association between *ORC6* expression level and immune cell infiltration. We identified a positive correlation between *ORC6* expression and the immune infiltration level of CD8 + T-cells in tumors of KIRC and UVM, while a statistical negative correlation between *ORC6* expression and the immune infiltration level of cancer-associated fibroblasts, tumor endothelial cells, and Treg cells in certain tumors by means of multiple immune deconvolution methods. Previous studies have demonstrated that immune cell infiltration may contribute to tumorigenesis, development, and metastasis [47–49]. Cancer-associated fibroblasts are the most abundant cancer stromal cells that induce tumor cell proliferation, therapeutic resistance and immune exclusion [50, 51]. Tumor endothelial cells play a crucial role in tumor angiogenesis and the suppression of T cells in the tumor environment [52, 53]. Treg cells can inhibit T cell proliferation and secrete immunomodulatory cytokines [54]. Finally, CD8^+^ T cells function as killer cells that dominate antitumor immune responses and greatly influence the outcome of cancer immunotherapy [55]. The pan-cancer analysis revealed differences in correlation between ORC6 and infiltration of different types of immune cells.

Of special note, our data, for the first time to the best of our knowledge, demonstrated that the ORC6 is associated to Treg cell infiltration in prostate cancer. This effect might be attributed to the enhanced differentiation of naive CD4 + T cells to Treg cells [56], which was supported by our results of IHC staining. Also, such a sophisticated mechanism may also involve the altered AR’s role as licensing factor during the entire cell cycle progression be inhibit the AR mechanism, which is open to be investigated in the future studies. Furthermore, our data also showed that the *ORC6* expression level is positively correlated with immunosuppressive and immunostimulatory genes across cancers, hinting that ORC6 may act as a potential immune checkpoint. Altogether, ORC6 may be a potential target for immunotherapy, which needs to be enlightened with further preclinical investigations.

Several research significances and values of this study are worth being highlighted. Firstly, ORC6 plays an important role in tumorigenesis and may work as an independent prognostic biomarker for many types of cancers. Secondly, we found ORC6 may affect genetic stability by regulating MMR pathways and genes. Thirdly, ORC6 was identified to influence the tumor immune microenvironment by adjusting the immune cell infiltration. Finally, ORC6 may tune the therapeutic outcome of immunotherapy via regulating immunomodulatory gene expression across cancers. Meanwhile, further in-depth investigations based on the data from the present study are needed to explore the sophisticated functions of ORC6 and its relevant molecular mechanism in individual cancer.

In conclusion, this pan-cancer analysis comprehensively identified that the high expression of ORC6 predicts a poor prognosis, ORC6 participates in the MMR process, and ORC6 is correlated with immunomodulatory cells, cytokines, and genes. Our results prove that ORC6 might be a promising prognostic biomarker and an immunotherapeutic target for multiple cancers, especially prostate adenocarcinoma.

## Supplementary Information

**Additional file 1: Supplementary Figure 1.** Pan-cancer paired ORC6 mRNA expression level. **Supplementary Figure 2.** Genetic Alterations of *ORC6* in pan-cancer. **Supplementary Figure 3.** Protein expression, subcellular locations, and protein–protein interaction (PPI) network of ORC6. **Supplementary Figure 4.** Immunohistochemical staining results of ORC6 in indicated tumor tissues and normal tissues from Human Protein Atlas. **Supplementary Figure 5.** Disease-specific survival (DSS) Analysis dependent on ORC6 expression. **Supplementary Figure 6.** Correlation between ORC6 expression and disease-free interval (DFI). **Supplementary Figure 7. **Correlation between ORC6 expression and progression-free interval (PFI). **Supplementary Figure 8.** Gene set enrichment analysis (GSEA) of ORC6 across cancers. **Supplementary Figure 9.** Association between *ORC6 *expression level and immune subtypes across tumors. **Supplementary Figure 10.** Correlation between ORC6 expression and immunostimulatory genes across cancers in The Cancer Genome Atlas.

## Data Availability

The datasets used and/or analysed during the current study are available from the corresponding author on reasonable request.
