# Supplementary material for: ORC6, a novel prognostic biomarker, correlates with T regulatory cell infiltration in prostate adenocarcinoma: a pan-cancer analysis
Source: BMC Cancer. 2023 Mar 29;23:285. doi: 10.1186/s12885-023-10763-z (PMC10053432; doi:10.1186/s12885-023-10763-z)
Supplement: Supplementary file 1 — Additional file 1: Supplementary Figure 1. Pan-cancer paired ORC6 mRNA expression level. Supplementary Figure 2. Genetic Alterations of ORC6 in pan-cancer. Supplementary Figure 3. Protein expression, subcellular locations, and protein–protein interaction (PPI) network of ORC6. Supplementary Figure 4. Immunohistochemical staining results of ORC6 in indicated tumor tissues and normal tissues from Human Protein Atlas. Supplementary Figure 5. Disease-specific survival (DSS) Analysis dependent on ORC6 expression. Supplementary Figure 6. Correlation between ORC6 expression and disease-free interval (DFI). Supplementary Figure 7. Correlation between ORC6 expression and progression-free interval (PFI). Supplementary Figure 8. Gene set enrichment analysis (GSEA) of ORC6 across cancers. Supplementary Figure 9. Association between ORC6 expression level and immune subtypes across tumors. Supplementary Figure 10. Correlation between ORC6 expression and immunostimulatory genes across cancers in The Cancer Genome Atlas. [file 12885_2023_10763_MOESM1_ESM.docx]

Supplementary Material

# Supplementary Figures


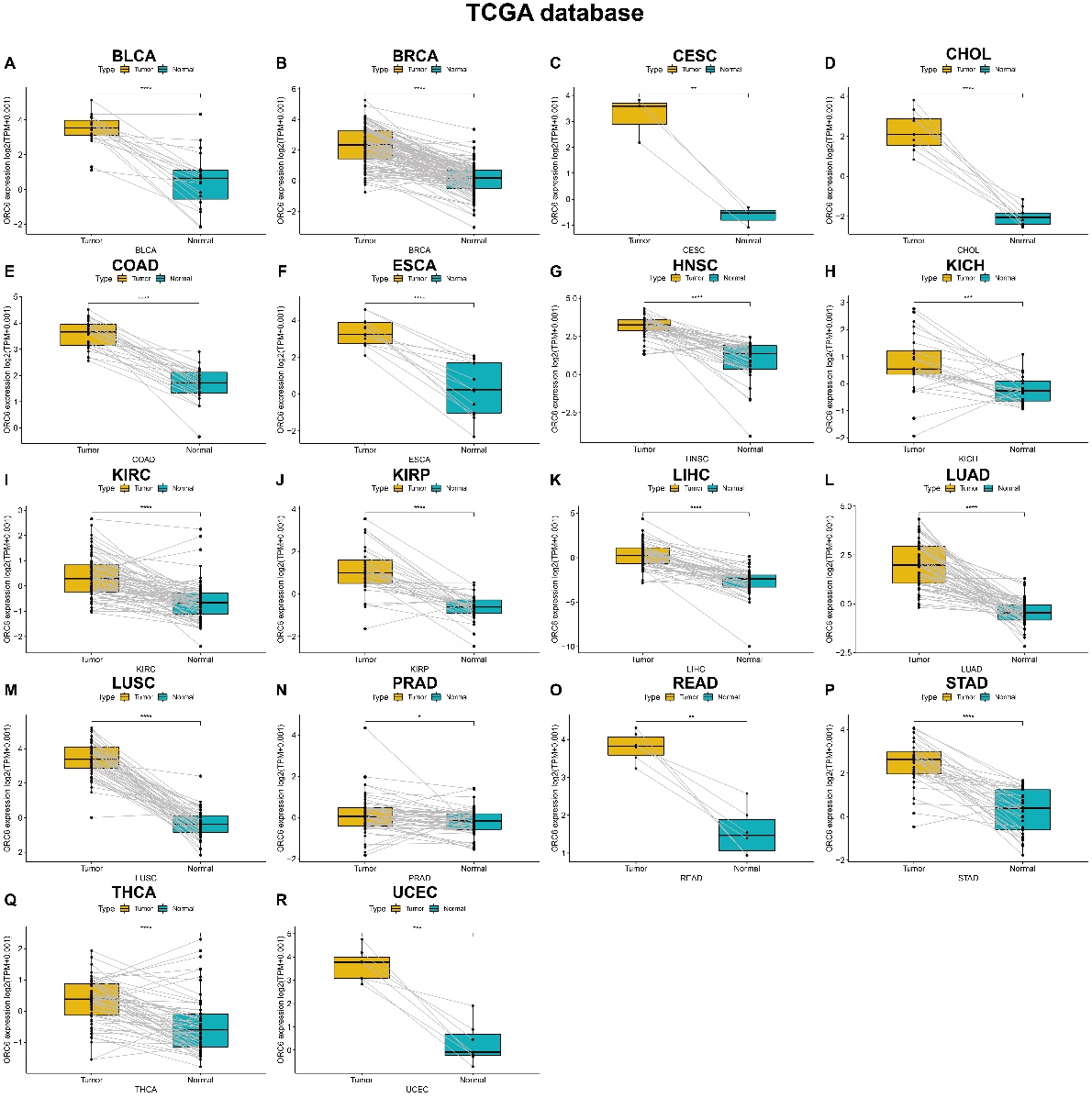


**Supplementary Figure 1.** **Pan-cancer paired ORC6 mRNA expression level. (A–R)** ORC6 mRNA expression level in tumor tissues and corresponding normal adjacent tissues from TCGA database. *p < 0.05, **p < 0.01, ***p < 0.001, ****p < 0.0001. Statistically non-significant results are not shown.

**
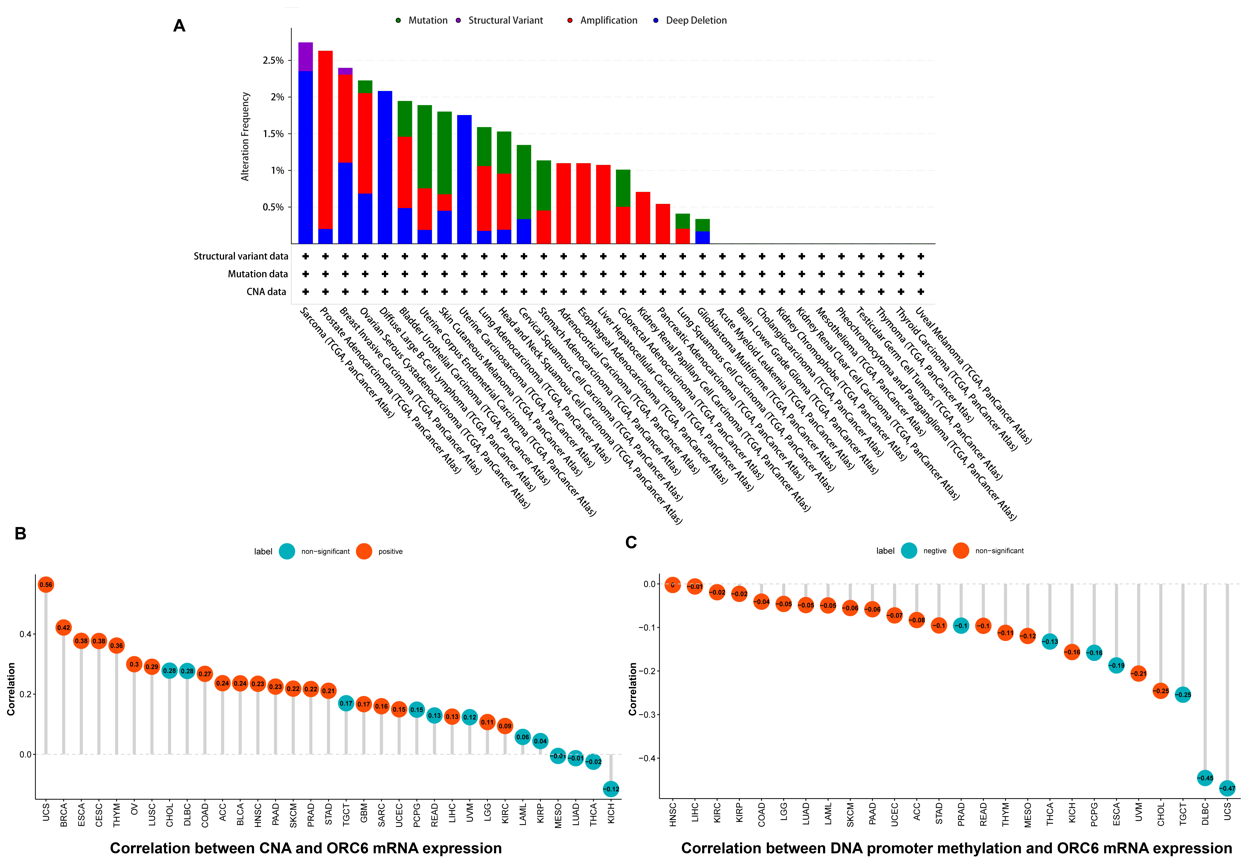
Supplementary Figure 2.** **Genetic Alterations of *ORC6* in pan-cancer. (A)** The alteration frequency with mutation type of *ORC6* using cBioPortal database. **(B)** Correlation between CNA and *ORC6* mRNA expression. **(C)** Correlation between DNA promoter methylation and *ORC6* mRNA expression.


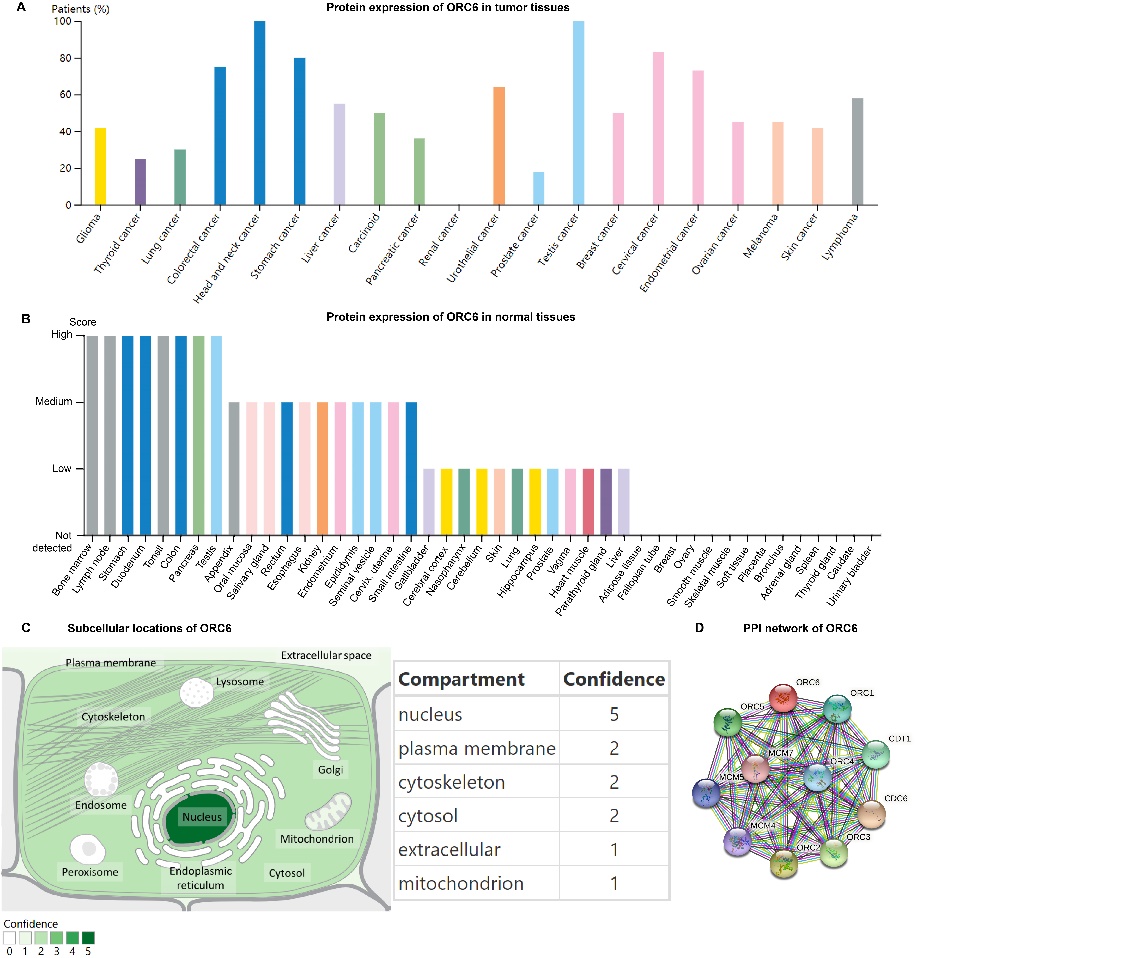


**Supplementary Figure 3. Protein expression, subcellular locations, and protein–protein interaction (PPI) network of ORC6. (A)** ORC6 protein expression in various tumor tissues using IHC staining data from the HPA database. **(B)** ORC6 protein expression level in indicated normal tissues using IHC staining data from the HPA database. **(C)** Subcellular locations of ORC6. **(D)** PPI network of ORC6 using pan-cancer data obtained from STRING.


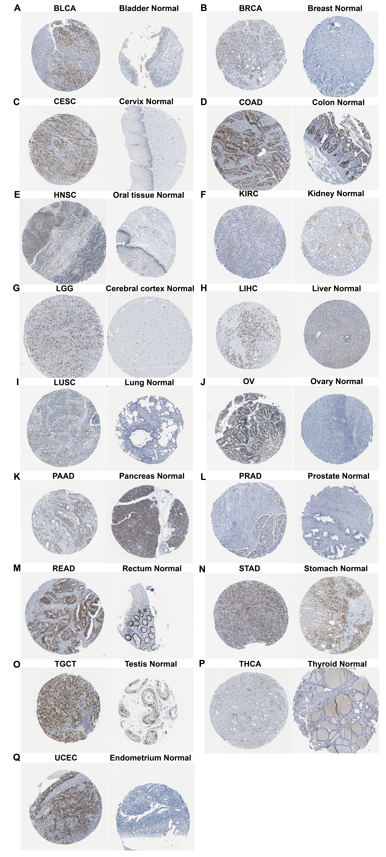


**Supplementary Figure 4. Immunohistochemical staining results of ORC6 in indicated tumor tissues and normal tissues from Human Protein Atlas.**

**
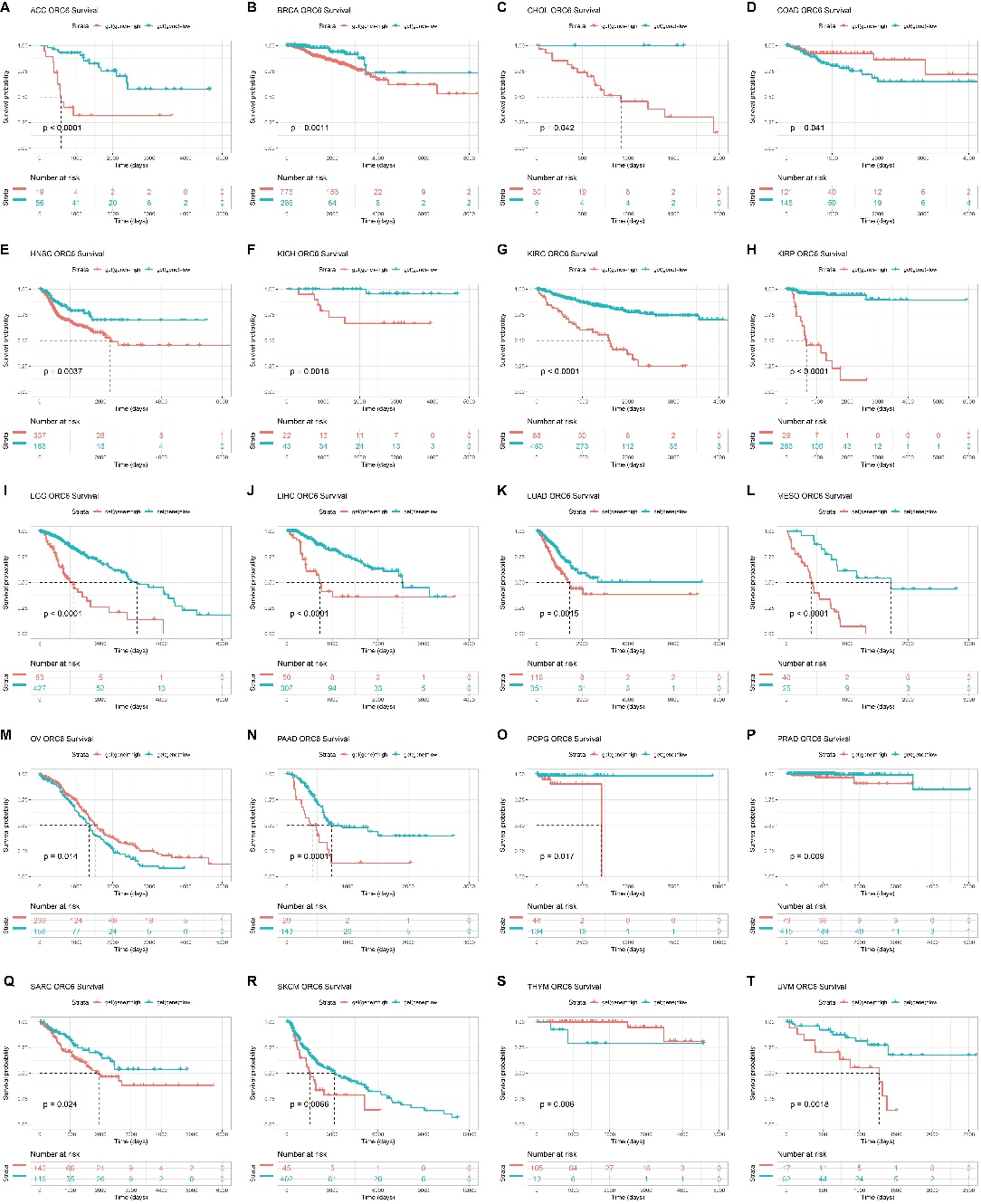
**

**Supplementary Figure 5.** **Disease-specific survival (DSS) Analysis dependent on ORC6 expression. (A–T)** Kaplan-Meier curves of DSS in diverse types of cancers from TCGA database. Statistically non-significant results are not shown.

**
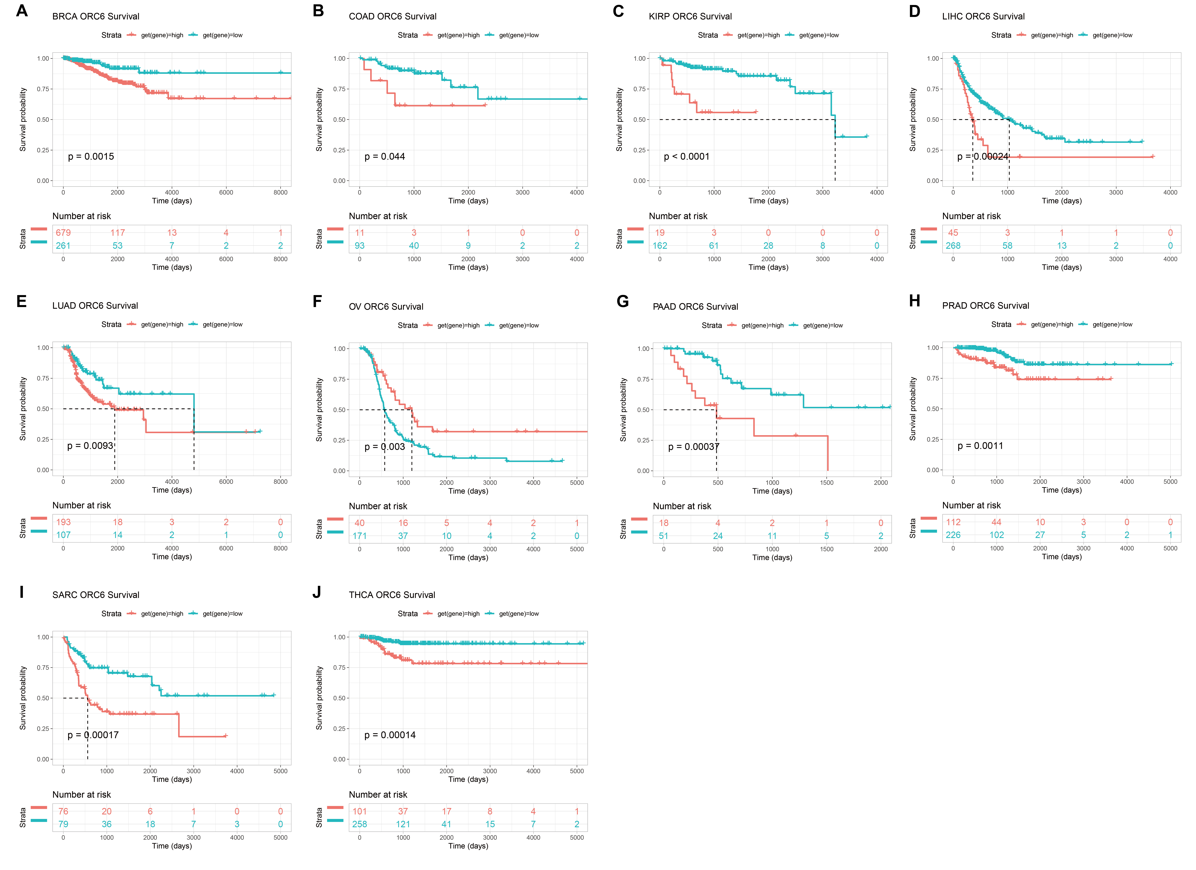
Supplementary Figure 6. Correlation between ORC6 expression and disease-free interval (DFI).** **(A–W)** Kaplan-Meier curves of DFI in 33 The Cancer Genome Atlas tumor types. Statistically non-significant results are not shown.

**
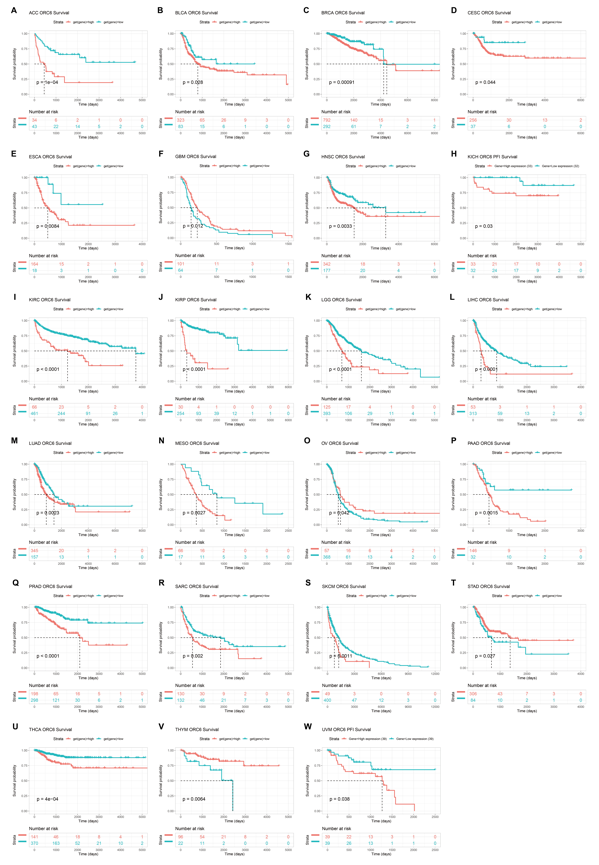
**

**Supplementary Figure 7. Correlation between ORC6 expression and progression-free interval (PFI).** **(A–J)** Kaplan-Meier curves of PFI in 33 The Cancer Genome Atlas tumor types. Statistically non-significant results are not shown.

**
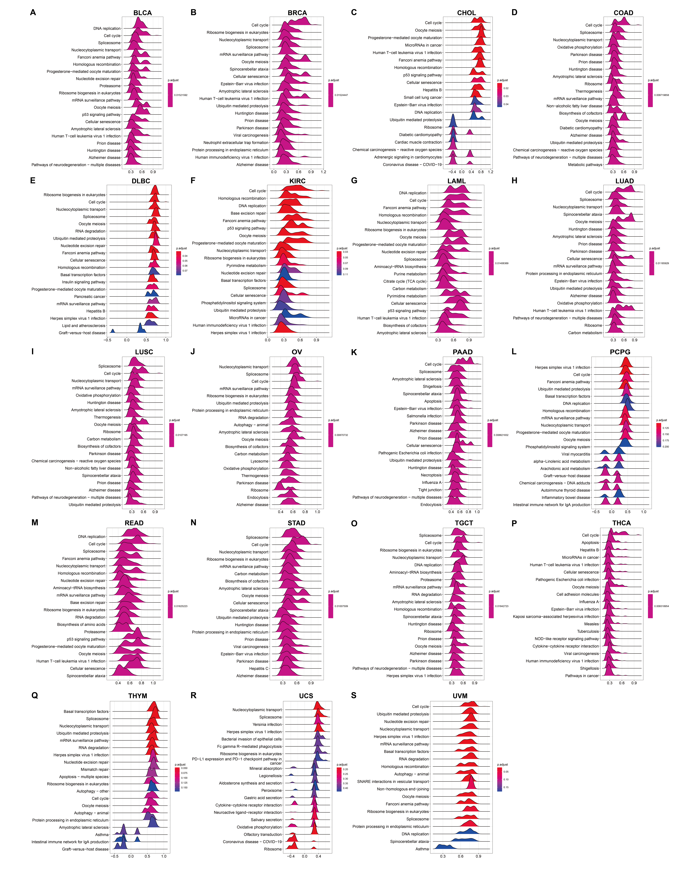
**

**Supplementary Figure 8. Gene set enrichment analysis (GSEA) of ORC6 across cancers. (A–S)** Kyoto Encyclopedia of Genes and Genomes results of *ORC6* GSEA in indicated tumor types using The Cancer Genome Atlas pan-cancer data.


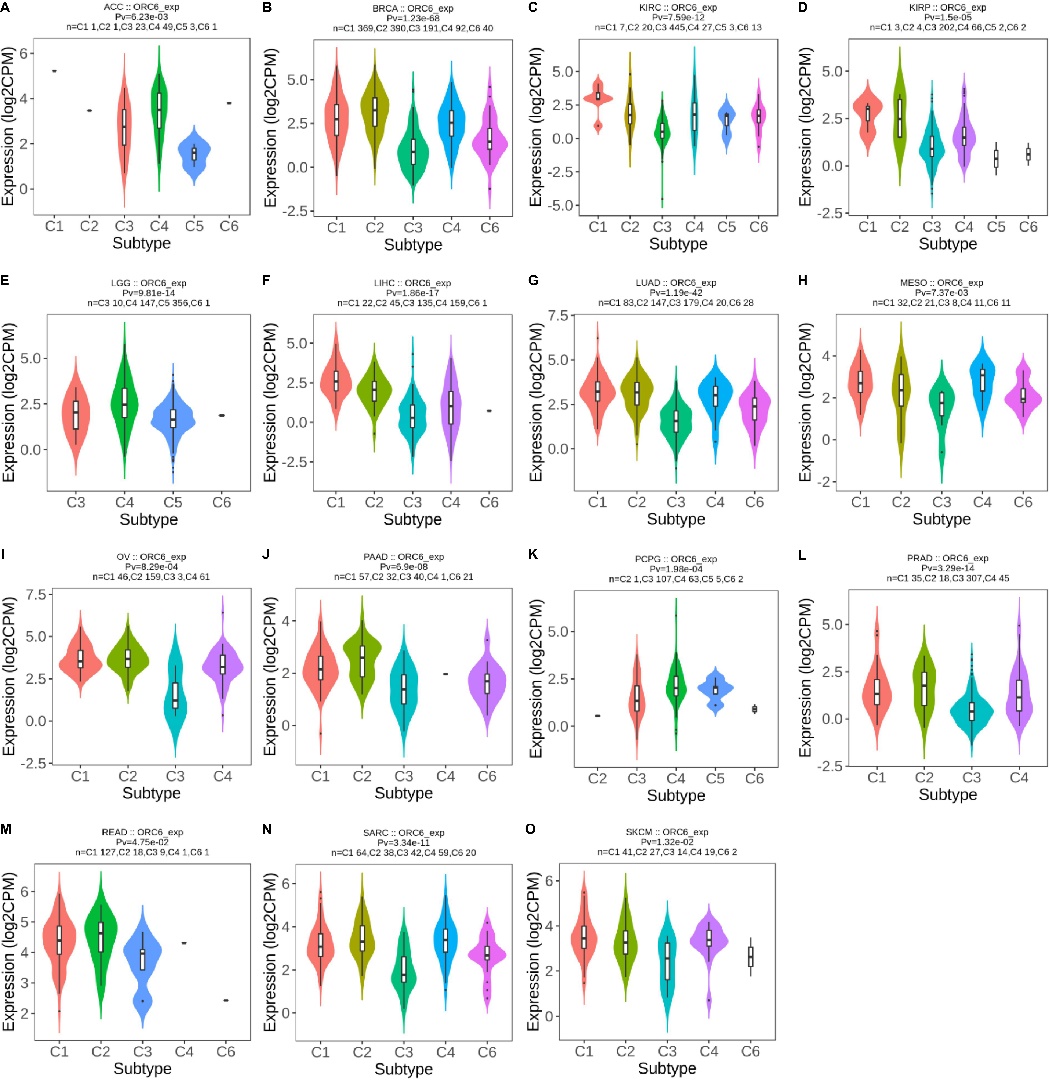


**Supplementary Figure 9. Association between *ORC6* expression level and immune subtypes across tumors. (A-O)** *ORC6* expression level in different immune subtypes using TISIDB database. Statistically non-significant results are not shown.


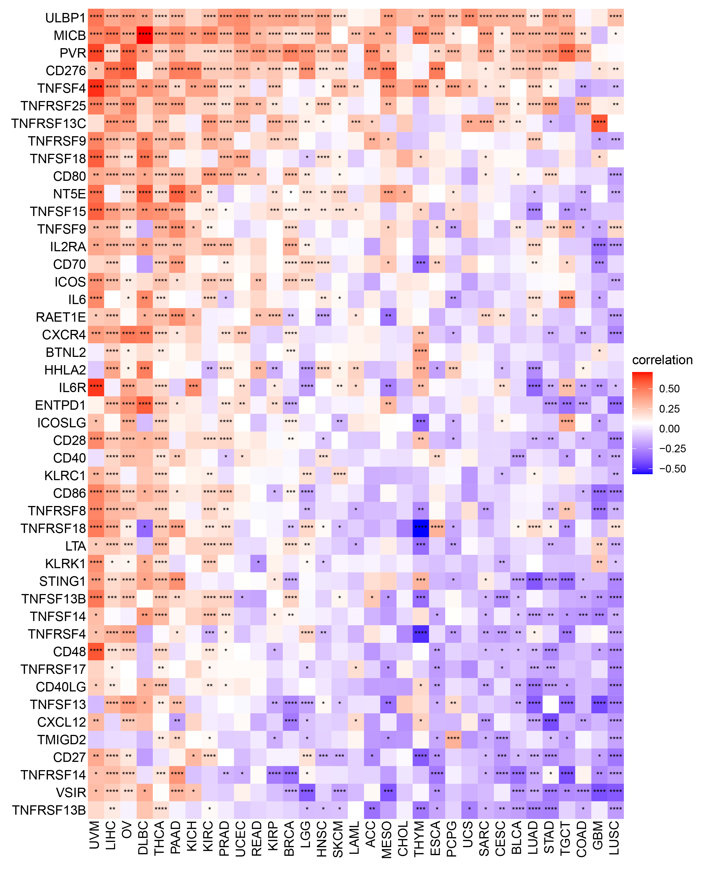


**Supplementary Figure 10. Correlation between ORC6 expression and immunostimulatory genes across cancers in The Cancer Genome Atlas.**
